# Achievement of long-term remission of disseminated histoplasmosis in an AIDS patient

**DOI:** 10.1016/j.mmcr.2019.12.012

**Published:** 2019-12-19

**Authors:** Akihide Nakamura, Isao Tawara, Kazuko Ino, Takeshi Matsumoto, Akinobu Hayashi, Hiroshi Imai, Yasunori Muraosa, Katsuhiko Kamei, Naoyuki Katayama

**Affiliations:** a Department of Hematology and Oncology, Suzuka General Hospital, 1275-53 Yasuzukacho-yamanohana, Suzuka, Mie, 513-8630, Japan; b Department of Hematology and Oncology, Mie University Graduate School of Medicine, Tsu, Mie, 514-8507, Japan; c Department of Transfusion Medicine and Cell Therapy, Mie University Hospital, Tsu, Mie, 514-8507, Japan; d Pathology Division, Mie University Hospital, Tsu, Mie, 514-8507, Japan; e Medical Mycology Research Center, Chiba University, Chiba, Chiba, 260-8673, Japan

**Keywords:** Disseminated histoplasmosis, *Histoplasma capsulatum*, HIV/AIDS

## Abstract

Histoplasmosis, a fungal infection caused by *Histoplasma capsulatum*, is poor prognosis once it disseminated, especially in immunocompromised patients. A 50-year-old Japanese-Brazilian male with multiple cervical lymphadenopathies was diagnosed as disseminated histoplasmosis and acquired immunodeficiency syndrome (AIDS). Anti-fungal therapy was initiated followed by anti-retroviral therapy (ART). He achieved long-term remission by treatment with voriconazole. Here we report a case of an AIDS patient with disseminated histoplasmosis who achieved long-term survival in non-endemic area.

## 1 Introduction

Histoplasmosis, a fungal infection caused by *Histoplasma capsulatum*, (*H. capsulatum*), is endemic in some areas of Latin American, African and Asian continents, but it is rare in Japan [1,2]. The prognosis of disseminated histoplasmosis in AIDS patients is generally poor and it is one of the major causes of death of AIDS patients [3]. Recent reports suggest that reducing delays of diagnosis and therapy initiation leads to improvement of prognosis of disseminated histoplasmosis in AIDS patients [4,5]. Polymerase chain reaction (PCR) is highly sensitive method for diagnosis of histoplasmosis [6]. To improve the prognosis disseminated histoplasmosis patient, rapid and accurate diagnostic system of histoplasmosis is needed around the world even in non-endemic areas. We report herein a successful treatment case of an AIDS patient with disseminated histoplasmosis, initially suspected of AIDS-associated lymphoma in non-endemic area, Japan.

## 2 Case

A 50-year-old Japanese-Brazilian male with persistent fever, fatigue and multiple cervical lymphadenopathies consulted his primary care physician (Day −14). He was referred to regional hospital (Day −1) because of laboratory tests showed anemia, malnutrition, elevated LDH and CRP. Computed tomography (CT) at the regional hospital confirmed systemic lymphadenopathies and hepatosplenomegaly. He was suspected of malignant lymphoma and referred again to our hospital for further examination and treatment (Day 1).

Physical examination revealed multiple cervical lymphadenopathies and hepatosplenomegaly. Laboratory test showed bicytopenia (Hgb: 11.2 g/dL, PLT: 93,000/μL), elevated LDH (642 IU/L) and CRP (15 mg/dL), and disseminated intravascular coagulation (DIC). In addition, CD4-positive lymphocyte count was very low (3 cells/μL) and anti-human immunodeficiency virus (HIV) antibody was positive (Table 1). He had never known his HIV infection until the admission.

**Table 1:** Lab data.

Laboratoy Data					
Complete Blood Count			Coagulation		
WBC	5670	/μL	APTT	32.2	sec
Neu	5329	/μL	PT	14.5	sec
Lymp	56	/μL	Fib	346	mg/dL
CD4^+^ lymph	3	/μL	D-Dimer	48.61	μg/mL
Hgb	11	g/dL	FDP	59.1	μg/mL
PLT	93	x10^3/μL			
Biochemistry			Others		
TP	7.3	g/dL	HBs-Ag		Negative
Alb	2.5	g/dL	HCV-Ab		Negative
T-Bil	0.4	mg/dL	HIV-Ab		Positive
AST	69	U/L	TP-Ab		Positive
ALT	38	U/L	RPR		Positive
ALP	345	U/L	Asp-Ag	>5.0	
LDH	642	U/L	CMV C7-HRP	18/77800	
BUN	11.0	mg/dL	(1,3)-β-D glucan	7.5	pg/mL
Cre	0.79	mg/dL	PCT	0.23	ng/mL
Fe	19	μg/mL	IgG	2082	mg/mL
UIBC	121	μg/dL	IgA	1461	mg/mL
Ferritin	5455	ng/mL	IgM	102	mg/mL
Na	133	mmol/L	sIL-2R	4426	U/mL
K	3.3	mmol/L			
Cl	103	mmol/L			
Ca	10.3	mg/dL			
CRP	15.73	mg/dL			

WBC; white blood count, Neu; neutrophil, HGB; hemoglobin, PLT; platelet, TP; total protein, Alb; Albumin, AST; aspartate aminotransferase, ALT; alanine aminotransferase, ALP; alkaline phosphatase, LDH; lactate dehydrogenase, BUN; blood urea nitrogen, Cre; creatinine, Fe; Ferrum, CRP; C-reactive protein, APTT; activated partial thromboplastin time, PT; prothrombin time, Fib; fibrinogen, D-DM; d-dimer, FDP; fibrinogen/fibrin degradation products, HBs-Ag; hepatitis B surface antigen, HCV-Ab; hepatitis C virus antibody, HIV-Ab; human immunodeficiency virus antibody, TP-Ab; Treponema pallidum antibody, RPR; rapid plasma reagin test, Asp-Ag; Aspergillus antigen, PCT; Procalcitonin, sIL-2R; soluble interleukin-2 receptor.

**Fig. 1:**
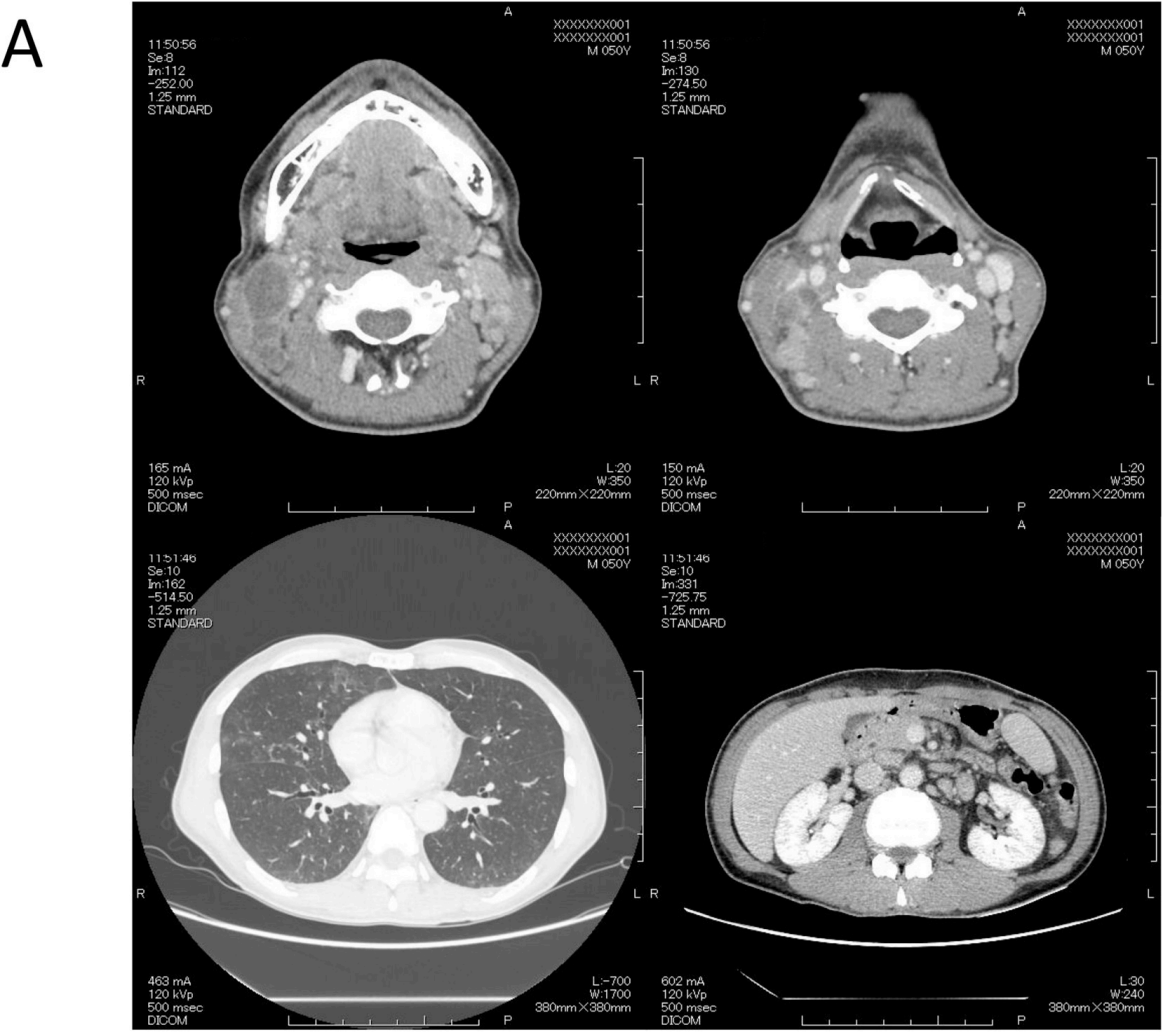

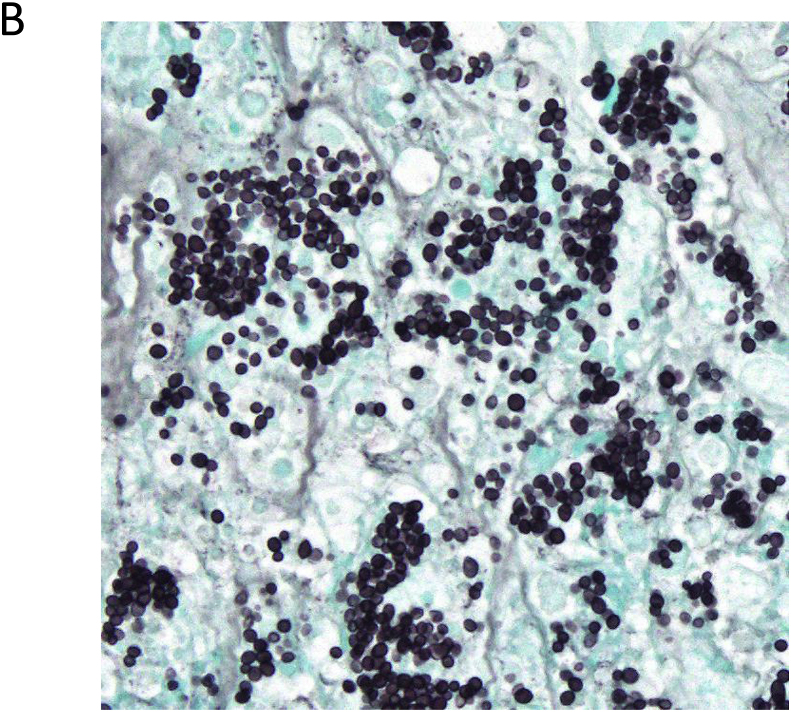
Computed tomography confirmed systemic lymphadenopathies, hepatosplenomegaly and ground glass opacity in both lungs (A) and fungal infection was suggested in cervical lymph node (Groccot stain, x40) (B).

The patient was hospitalized and enhanced computed tomography confirmed systemic lymphadenopathies, hepatosplenomegaly and ground glass opacity in both lungs (Fig. 1A). He was suspected of AIDS-associated lymphoma and virus-associated hemophagocytic syndrome (VAHS). Based on the laboratory findings on admission, anti-coagulation, bacterial (Levofloxacin; 500 mg QD, Azithromycin 1200 mg QW and Sulfamethoxazole/Trimethoprim; 400 mg/80 mg QD), viral (Valganciclovir; 900 mg QD) and fungal (Fluconazole; 100 mg QD) therapies were initiated. After improvement of DIC, bone marrow aspiration/biopsy and cervical lymph node biopsy were performed (Day 5).

The lymph node was necrotic and infiltrated plasma cells and histiocytes were confirmed (Day 7). And yeast-like fungi were also confirmed in those histiocytes. Clinicopathological findings suggested fungal infection such as coccidioidomycosis, paracoccidioidomycosis, toxoplasmosis, histoplasmosis. The pathogen was finally identified in *H. capsulatum* by pan-fungal PCR and DNA sequencing of the PCR product with 100% compatibility (Fig. 1B).

**Fig. 2:**
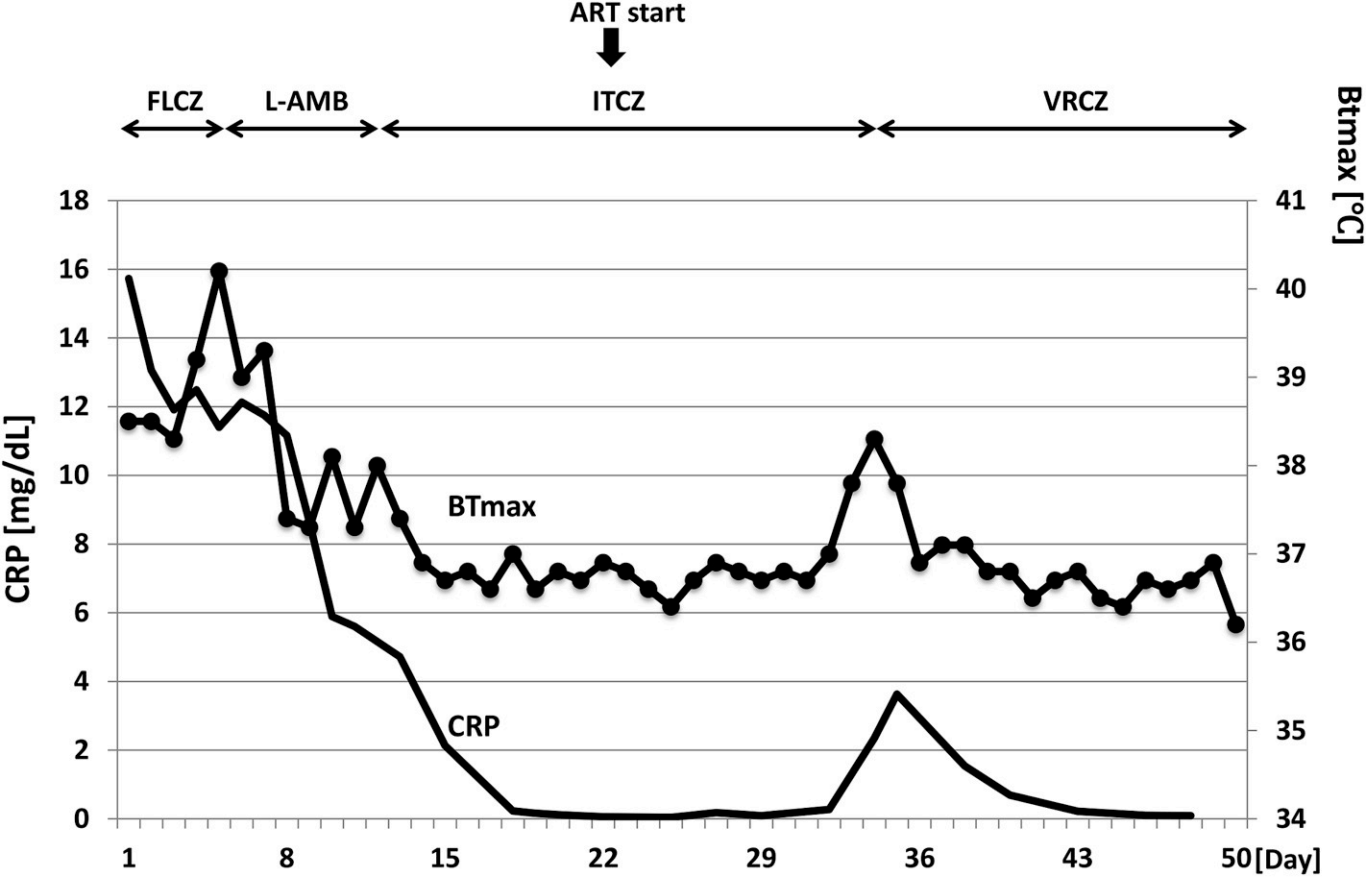
Clinical course of the hospitalized period. Changes of maximum body temperature in each day (BTmax) and CRP are shown. Antifungal therapy and timing of anti-retroviral therapy (ART) start are indicated on the top. FLCZ; fluconazole, L-AMB; liposomal amphotericin B, ITCZ; itraconazole, VRCZ; voriconazole.

**Fig. 3:**
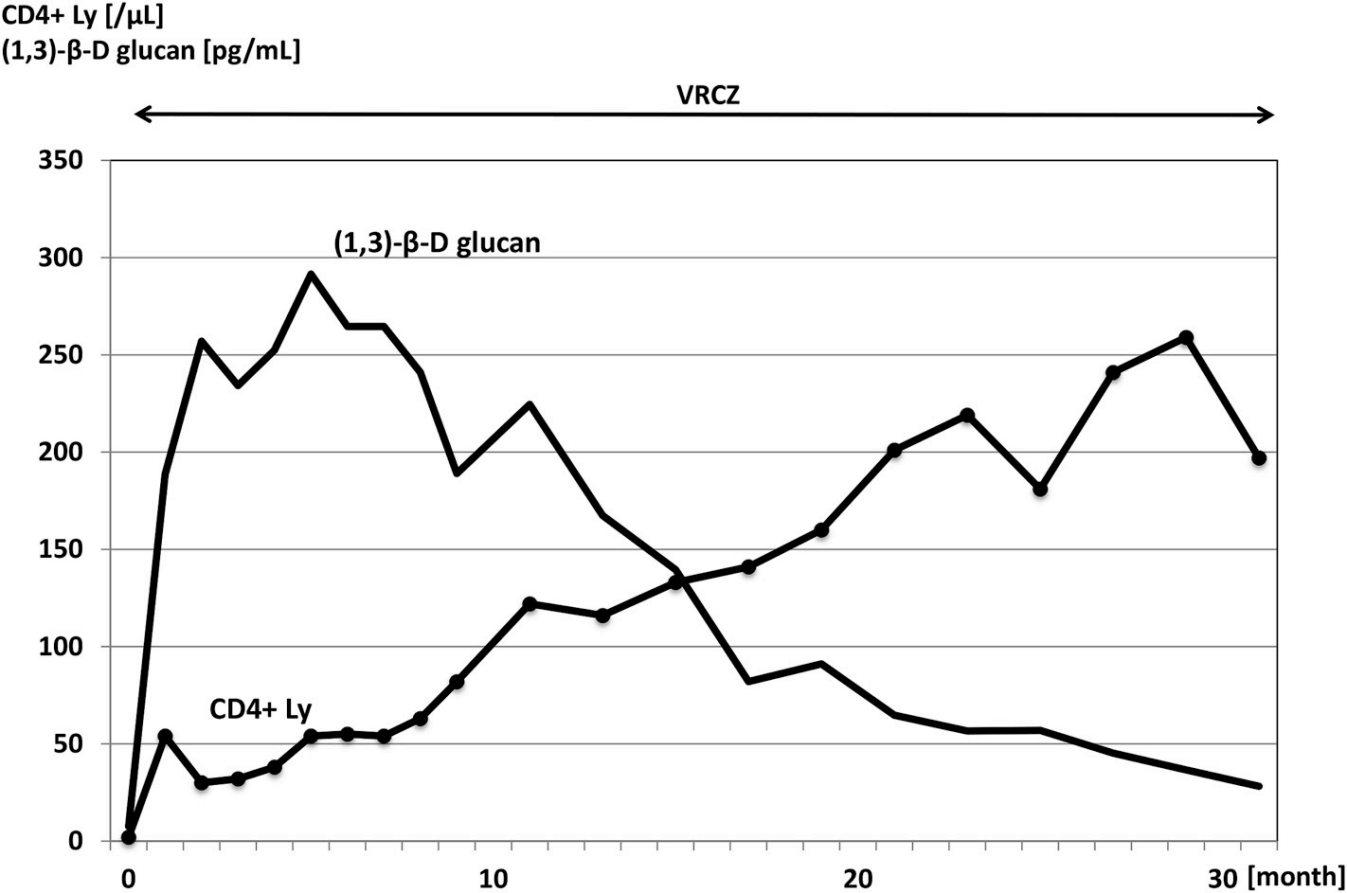
Clinical course of the 30 months. Changes of CD4-positive lymphocyte count (CD4 Ly) and level of (1,3)-β-D glucan are shown. Patient has been receiving voriconazole combined with ART for almost 30 months.

Treatment was initiated with 3 mg/kg/day intravenous liposomal amphotericin B (L-AMB) (Day 7), which resulted in clinical and biological improvement. However, antifungal treatment was switched from L-AMB to itraconazole (ITCZ) later (Day 18) because of renal dysfunction and hyponatremia. At the timing of anti-fungal treatment switch, antiretroviral therapy (ART) with dolutegravir/abacavir/lamivudine was initiated (Day 24). However, ITCZ was switched to voriconazole (VRCZ) (Day 34) because of marked elevation in CRP and (1,3)-β-D glucan. VRCZ treatment improved the patient's condition and he was discharged from the hospital (Day 50) (Fig. 2). He has been receiving VRCZ combined with ART for 2.5 years as an outpatient (Fig. 3). Although (1,3)-β-D glucan is still positive, he has no symptoms and no lymphadenopathies was pointed out on computed tomography.

## 3 Discussion

Histoplasmosis is a mycosis caused by *H. capsulatam*. It is commonly found in endemic areas such as Latin American, African and Asian continents. But Japan is known to be non-endemic area. The patient had travel history to endemic area several years before onset of histoplasmosis.

Histoplasmosis is self-limited and not fatal in immunocompetent hosts [7]. But once *H. capsulatum* disseminates in immunocompromised patients such as untreated HIV infection, tuberculosis and advanced cancer, it will be life-threatening infections [8]. In the present case, the patient had only 3 cells/μL of CD4-positive lymphocytes and supposed to be severe immunodeficiency.

Disseminated histoplasmosis is normally treated with L-AMB or ITCZ [2]. Response rate is 80–100% with ITCZ without CNS invasion and duration of treatment is at least one year [2]. In an immunocompromised patient, treatment should be continued until recovery of immunity, clinical improvement and biological resolution. In the present case, the patient was initially treated with L-AMB (started at 3 mg/kg/day). However antifungal treatment was switched from L-AMB to ITCZ (200 mg/day) two weeks later because renal dysfunction and hyponatremia occurred. ITCZ was switched again to VRCZ later because of marked elevation in CRP and (1,3)-β-D glucan. It might be possible that the level of ITCZ was below the effective blood concentration. He was discharged from our hospital 50 days after admission. He has been receiving VRCZ combined with ART as an outpatient with clinical remission.

It is suggested that reducing delays of diagnosis and therapy initiation leads to improvement of prognosis of disseminated histoplasmosis in AIDS patients. Although it took several weeks to be fixed antifungal agents in the present case, rapid diagnosis and initiation of treatment lead to successful control of disseminated histoplasmosis in an AIDS patient.

In conclusion, we report the case of an AIDS patient with disseminated histoplasmosis who achieved long-term survival with antifungal therapy-combined ART. With increasing numbers of immunocompromised patients, caution should be exercised with rare endemic diseases even in non-endemic area.

## Declaration of competing interest

There are none.
